# The Influence of Family Crisis Coping Strategies on Family Quality of Life in the Assessment of Patients with Idiopathic Scoliosis

**DOI:** 10.3390/ijerph20021177

**Published:** 2023-01-09

**Authors:** Barbara Cyran-Grzebyk, Lidia Perenc, Justyna Wyszyńska, Gabriela Kołodziej-Lackorzyńska, Joanna Majewska

**Affiliations:** Department of Physiotherapy, Institute of Health Sciences, College of Medical Sciences, University of Rzeszów, Aleja Majora Wacława Kopisto 2A, 35-315 Rzeszów, Poland

**Keywords:** idiopathic scoliosis, family crisis, mental health, family quality of life

## Abstract

The aim of the study was to assess the impact of the strategies of coping with the family crisis in children and adolescents with idiopathic scoliosis on the level of their family’s quality of life (FQOL). The study group consisted of 100 adolescents (girls 83%, boys 17% of the entire main group) with an average age of 14 (13.83 ± 1.92). The control group consisted of the same number of people as the study group (girls 78%, boys 22% of the entire control group) with an average age of 14 (14.09 ± 2.16). The FQOL scale was used to assess the family’s quality of life, and the F-COPES scale was used to assess the problem-solving strategies used by the family (individual members) in a difficult life situation. Statistical analysis showed significant differences between the two compared groups. While dealing with a crisis situation in their families, adolescents treated for idiopathic scoliosis were using the spiritual support strategy (M ± SD 5.12 ± 2.07) significantly more often, while their healthy peers much more willingly and more often benefited from other possible strategies (F-COPES). Additionally, statistically significant differences were observed in the assessment of children and adolescents from both groups that the usage of different strategies available on the F-COPES scale correlated and affected the areas of the FQOL level of their families. Gaining spiritual support had a negative impact on the area of physical and financial well-being of FQOL, as assessed by juveniles with SI (R = −0.254, *p* = 0.011). However, in the opinion of healthy peers, the strategy of gaining social support (F-COPES) resulted in a higher level of FQOL in the area of disability problems (*p* = 0.005). A long process of SI treatment can cause crisis situations for patients and their families and affect both the physical and mental health of patients by changing the FQOL level of their families.

## 1. Introduction

Idiopathic scoliosis (SI) is the most common spine disorder in adolescence. It affects approximately 2% to 4% of adolescents in Europe and 1% to 3% in the US. It is slightly more common in girls than boys. Female gender is characterized by up to 10 times higher risk of curvature progression. Most adolescents with SI do not experience clinical symptoms of pain, but the disease can lead to rib deformities and respiratory dysfunction, and can cause serious cosmetic problems, distress to sufferers, and mental health problems [1,2]. An additional negative factor is the prospect of long-term conservative or surgical treatment. The whole family, mainly parents, is involved in this sometimes-complex process of treating a child with SI. The patient’s family is actively introduced to the treatment process and becomes an integral part of it. This fact can sometimes cause crises in the family system, which had previously functioned in a different way. The appearance of the disease disrupts its functioning and affects the quality of life of the whole family [3,4].

We can talk about a crisis in the family on the professional, financial, and axiological level. It may also concern, as in this case, health or the discussed sphere of the family. Usually associated with some kind of threat, deterioration, or even deadlock, crisis is undoubtedly a very difficult and specific situation that disturbs the proper functioning of, for example, a family. It lasts until all involved in it get used to the changes and adapt to new conditions to restore stability and balance in the family. The available literature describes several types of family crises: normative developmental crisis (the effect of going through typical family development phases), random, situational, incidental (caused by unpredictable events, e.g., illness), and endogenous (pathological family patterns, e.g., alcoholism). A crisis occurs when, despite the applied strategy, it is not possible to restore the current balance, when the achieved balance turns out to be unstable in the face of new, difficult challenges. There are many factors affecting the family’s ability to cope with critical situations (such as support from friends and community, age, family seniority, financial stability, religiousness, etc.) [5].

The World Health Organization (WHO) defines quality of life (QOL) as “a comprehensive way of evaluating by an individual his or her physical and emotional health, independence in life and degree of dependence on the environment, as well as relationships with the environment, personal beliefs and convictions”. The QOL assessment suggests a subjective evaluation of human life as a whole or its components, such as social contacts, financial security, or satisfaction with the performed profession/activity [6]. Each patient struggling with a chronic disease, in this case with scoliosis, functioning in a given family system in a stressful and difficult situation (caused, for example, by deformation of the trunk), uses various strategies to cope with a family crisis, and as a consequence may experience deterioration of physical and mental health. An important aspect of SI treatment and the priority goal of conservative treatment is to stop the progression of curvature, although other important goals of therapy, i.e., improving the quality of life of patients and their families, may have a positive impact on the mental health of patients [7,8]. It is worth asking the question as to whether the use of family crisis coping strategies affects the FQOL of the patient. In order to be able to better select therapeutic goals, assess the risk of progression, and understand the mental health problems of patients with SI, it is reasonable to evaluate the above strategies and their impact on the FQOL of patients treated for SI.

During the long, often long-term treatment process of patients with scoliosis, there is a need to combine conservative treatment, including scoliosis-specific physiotherapeutic exercises (PSSE), including daily living activities (ADL) with back brace treatment. Corset treatment is an additional negative factor that can reduce frame of mind and self-esteem of an adolescent patient [9,10]. Other factors that bother juveniles with SI are the need to regularly perform corrective exercises and constant self-control of posture, as well as constant control by parents in terms of performing assigned physical activities and wearing a corset, which can cause stress, anger, depressive mood, disputes in the family and, consequently, reduce the general FQOL level of their families. A person suffering from chronic diseases, functioning in the family system, requires support and encouragement from relatives (family), but also copes with the family crisis caused by the disease in an individualized way [11]. The F-COPES scale (Family Crisis Oriented Evaluation Scales F-COPES) can be used to assess the ways of coping with a family crisis in patients with SI. It allows for measuring what problem-solving strategies are being used by a patient in a difficult life situation, e.g., during treatment [12,13].

This scale shows how to get familiar with strategies of coping with the family crisis of patients whose physical and mental health changes as a result of the disease. As a result of crisis situations occurring in patients with SI and their families, the level of FQOL is also modified. There are a lack of available studies regarding the evaluation of F-COPES and its impact on FQOL. Thus, the aim of this study was to evaluate the impact of F-COPES assessed by children and adolescents with SI on the FQOL level of their families. For the purposes of the study, the F-COPES scale was assessed by children and adolescents, and the results are presented in this article.

The goal of the study was to examine and compare the types of strategies for coping with crisis situations occurring in the family system in the assessment of children and adolescents with SI (main group) and to compare them with the control group of peers without a diagnosis of SI (comparison group), as well as to assess the impact of the above strategies on individual FQOL areas of their families. It was assumed that there are differences in the strategies of coping with the family crisis used by children and adolescents from the main and comparative groups and that, in their opinion, individual strategies affect the level of FQOL.

## 2. Materials and Methods

### 2.1. Organization of Research

The analysis covered a total of 200 families of children and adolescents aged 7–18 from the Podkarpackie County (Poland) in 2019–2021. The study group consisted of 100 patients with SI with their families, and the control group of 100 peers without SI, also with their families. In both groups of children and adolescents, the female gender predominated. The selection of the sample was carried out in a non-random manner, and all participants that met the eligibility criteria qualified (recognition sample) [14]. Finally, the study group consisted of 100 patients aged 9 to 18, attending rehabilitation treatment due to SI at the Clinical Rehabilitation and Education Center at the County Clinical Hospital No. 2 in Rzeszów. The study utilized the following research tools: the author’s medical history and physical examination card created for the purpose of the study, the Family Crisis Oriented Evaluation Scales (F-COPES), and the Family Quality of Life Scale (FQOL). The examination was conducted once.

### 2.2. Group Characteristics

The criteria for inclusion in the study group were as follow: voluntary consent of a child or adolescent with SI to participate in the study, lack of other diseases or developmental defects, age between 7–18, two-parent family, diagnosed SI, current X-ray of the entire spine with the iliac plates in the frontal plane (not older than 6 months), active participation in conservative treatment—physiotherapy in a homogeneous manner according to PNF method (on average 45 min once a week, for at least 6 months) and written consent of the child’s parents (legal guardians) to participate in the study. The control group included children and adolescents without SI and other congenital defects, from two-parent families, after obtaining written consent to participate in the study granted by the parents or legal guardian. The exclusion criteria (both groups) were as follows: lack of voluntary/written consent of the child’s or adolescent’s parents (legal guardians) to participate in the study, age of the respondents being under 7 or over 18, single-parent family, the presence of diseases or developmental defects in the patient, lack of a current X-ray of the entire spine with the iliac plates in the frontal plane (older than 6 months), patients not undergoing physiotherapy treatment, patients recovering after spinal surgery, lack of cooperation with the patient and their family. Girls with SI constituted 83% of the entire study group, and boys constituted 17%, while girls without SI constituted 78% of the control group, and boys constituted 22%. The mean age of children and adolescents from both groups was 14. In both groups, children and adolescents were of similar age. The Cheneau corset was the only model worn by the patients. Detailed characteristics of the study and control groups are presented in Table 1.

### 2.3. Methods 

#### 2.3.1. Family Crisis Oriented Evaluation Scales (F-COPES)

The F-COPES scale enables us to assess the strategies for solving problems in a family in a difficult life situation. The scale sheet contains 30 items. The higher the score, the more often a specific strategy is used to solve crisis situations in the family. It is assumed that the more strategies a family uses in difficult situations, the better their ability to cope with a family crisis. The questionnaire diagnoses 5 strategies of action [15]:

1. seeking social support (9 items)—by choosing this strategy, the family actively seeks help from relatives, friends, and acquaintances from the social circle;
2. transformation of the meaning of the situation—redefinition of the situation (8 items)—it is based on giving a different, acceptable meaning to the stressful situation;
3. seeking spiritual support (4 items)—related to religious activity of people experiencing a stressful situation;
4. seeking outside help (5 items)—mobilizing family forces to seek and accept help from an organization or institution;
5. passive assessment of the situation (4 items)—this is the choice of a state of passive waiting, without one’s own involvement and activity aimed at any solution to settle difficult situations.

The strategies in points 1, 3, and 4 are considered the family’s external ways of coping. They show family systems that prefer a type of struggle involving the use of energy and support coming from outside. Strategies 2 and 5 are internal methods that mean that individual family members use the resources provided by the family system itself [16]. 

#### 2.3.2. The Family Quality of Life Scale (FQOL)

The FQOL questionnaire was used to assess the level of quality of life related to the health of the family and its individual members. The scale is used to assess family members’ perceptions of their satisfaction with various aspects of family quality of life and includes five subscales: family interaction, parenting, emotional well-being, physical and financial well-being, and support for people with disabilities. Respondents answered the questions according to standardized 5-point categories: very dissatisfied, dissatisfied, hard to say, satisfied, very satisfied. The scale form was completed by children and adolescents from both groups. The overall and sub-indexes of the FQOL questionnaire are positively oriented, meaning that a high score means a high level of FQOL. The questionnaire was based on the principle of providing answers from the perspective of the last year (last 12 months). The FQOL questionnaire was completed by both family members from the study group and the control group [17].

### 2.4. Statistical Analysis

The statistical analysis of the obtained results was performed using the R program, version 3.6.2 (Foundation for Statistical Computing, Vienna, Austria) [18]. The analysis of quantitative variables in both groups was performed using the mean (M), standard deviation (SD) and median. In turn, the analysis of qualitative variables was carried out by calculating the number and percentage values. The comparison of the values of qualitative variables in groups was made using the chi-square test (with Yates’ correction for 2 × 2 tables) or Fisher’s exact test where low expected frequencies appeared in the tables. The comparison of quantitative variables in the two groups was performed using the Mann–Whitney test (*p*). In order to identify statistically significant groups and detect statistically significant differences, a post-hoc analysis was performed using Dunn’s test. Correlations between quantitative variables were analyzed using Spearman’s correlation coefficient (R).

Multivariate analysis of the independent influence of many variables on the quantitative variable was performed using the linear regression method. The results are presented as regression model parameter values with a 95% confidence interval (95% CI). The level of statistical significance was assumed at *p* < 0.05. All *p* values below 0.05 were interpreted as indicative of significant relationships (*p* < 0.05).

## 3. Results

Comparative analysis (Table 2) of the results of the F-COPES scale of both groups (study and control) showed that children and adolescents from the study group more often sought spiritual support (*p* = 0.012), which emphasizes the importance of prayer, religious activity, and seeking help and advice from clergy for adolescents with SI. In the area of other strategies, patients with SI showed passivity, which could be associated with a sense of little influence on the course of the disease. The awareness that “I can’t do anything about it anyway” was often an excuse for the lack of initiative, relieved one from the attempts to solve problems caused by the presence of the disease in the family. While children and adolescents from the control group more often used coping strategies with family crisis, such as transforming the situation (*p* < 0.001), passive assessment of the situation (*p* < 0.001), gaining social support (*p* < 0.001), and relying on formal structures (*p* = 0.001). These results prove that healthy children and adolescents adopt an active attitude more often than patients treated for SI when faced with a stressful situation in the family. It can be associated with greater undertaken activity in the process of coping with this situation, contrary to juveniles with SI. Additionally, healthy adolescents, more often than those suffering from SI, tried to actively accept stressful situations occurring in the family. Internal strategies, i.e., transforming the meaning of the situation and passive assessment of the situation showed that the youth from the control group more often took an active attitude towards crisis situations in their families [19,20,21,22].

To our knowledge, the available literature lacks normative data on FQOL and F-COPES for healthy individuals. The ones that can be found most often concern research on the population of sick people. There are many works and studies on the validation of both questionnaires (different countries), although we have not come across any work on this topic concerning the population of healthy children.

In the study group, statistical analysis using Spearman’s rank correlation coefficient in the use of stress coping strategies such as transforming the meaning of the situation and passive evaluation of the situation (F-COPES) did not show any significant correlations with the level of FQOL according to the assessment of children and adolescents with SI (Table 3).

Gaining social support in the assessment of children and adolescents with SI correlated statistically significantly and negatively only with physical/financial well-being (FQOL). Children and adolescents with SI, who more often sought social support in solving family problems, assessed the physical and material well-being of the family at a lower level (R = −0.254, *p* = 0.011) (Table 4). 

In the evaluation of children with SI, using the help of other people and society from outside the family system as one of the strategies to fight the family crisis resulted in a decrease in the above-mentioned FQOL level of their families. The dependence showed that the introduction of the above-mentioned external factor into a functioning, relatively well-organized family system caused, in their opinion, a reduction and disturbance of the sense of satisfaction with the physical (health, physical condition), mental, and material state of the family’s life. On the other hand, based on the statistical analysis, there was no relationship between the acquisition of spiritual support and the level of FQOL in the assessment done by adolescents with SI.

The analysis using the linear regression model showed that a significant (*p* < 0.05) independent predictor of the FQOL level in the area of family interactions in the assessment of children and adolescents with SI was the use of transforming the situation and relying on formal structures as a strategy for coping with a family crisis (Table 5). Both the internal strategy, i.e., active acceptance of the difficult situation currently prevailing in the family of patients with SI, and the external strategy, i.e., using the help of various types of formal institutions, mainly health care facilities, contributed the greatest value and positive impact on the improvement of interactions in the family, as assessed by children from the study group. The control group has not indicated such a relationship. Therefore, it could be presumed that the illness of one of the family members, in their opinion, and the two ways of dealing with the family crisis related to it, increases the level of FQOL in the area of family correlations. On the other hand, an independent predictor of the level of FQOL in the area of physical/financial well-being was obtaining social support. Each additional point obtained in the assessment using the F-COPES scale reduced the level of FQOL in the area of physical/financial well-being by 0.134 points on average. Gaining social support (F-COPES) turned out to be a negative predictor. The statistical analysis showed that a statistically significant (*p* < 0.05) independent predictor of the FQOL level in the area of problems with disabilities was gaining social support. Each additional point obtained in the assessment using the F-COPES scale reduced the FQOL level in the area of disability problems by an average of 0.208 points.

In the assessment of juveniles from the control group, no statistically significant correlations (*p* ≥ 0.05) of transforming the meaning of the situation and passive assessment of the situation (F-COPES) with the level of FQOL were found. More frequent use of the strategy of gaining social support resulted in a higher level of FQOL in the area of disability problems (*p* = 0.005) in the assessment of children without SI (Table 6).

Gaining spiritual support had a significant impact on the FQOL level in the assessment of children and adolescents from the control group. The variable correlated significantly and positively with emotional well-being (R = 0.207, *p* = 0.039). More frequent use of this strategy increased the level of FQOL in the area of emotional well-being (Table 7). Statistical analysis showed no significant relationships (*p* ≥ 0.05).

Based on the linear regression model, it was shown (Table 8) that for seeking spiritual support (F-COPES), the regression parameter was 0.291. Each additional point obtained in the assessment using the F-COPES scale increased the level of FQOL in the area of spiritual support by an average of 0.291 points in the assessment of children without SI. In this case, this strategy was considered a positive predictor of the FQOL level in the assessment of juveniles without SI. The other two, gaining spiritual support and passive assessment of the situation, are considered negative predictors of the FQOL level.

## 4. Discussion

SI is defined as a chronic disease. The appearance of a child with idiopathic scoliosis in the family, which, as a consequence of the progression, affects the shape of the human body, becomes a cause of stress in patients. In addition, the prospect of a possible surgery in the treatment process becomes a negative factor affecting the physical and mental health of patients and their caregivers. [23,24,25]. Initially, the parents of a child diagnosed with SI start the procedure of seeking help for their offspring, and then introduce the patient to the treatment process. A difficult time for the whole family begins, in which the word scoliosis will be heard often. This is not an easy period for the child and individual family members, and it usually coincides with the moment of puberty, which concerns changes in the body structure, psyche, and behavior of the growing child [26]. On the basis of the conducted research, the existence of differences in the use of the family crisis coping strategy (F-COPES) by adolescents with scoliosis (the study group) and without scoliosis (the control group) was established. In their own research, Eliason and Richman presented developmental stages in adolescence and the impact of scoliosis and the results of its treatment on them. 

The authors pointed out that young people seek independence from adults and, at the same time, peer conformism. These tasks are difficult to achieve when the adolescent is forced to extend a period of dependence on parents and physicians for medical care, resulting in rebellion by non-compliance with the treatment procedure [27]. The stress of the patients causes crisis situations in their families and affects FQOL. On the basis of our own research, the existence of differences in the strategies of coping with a family crisis used by children and adolescents from the study group and the control group were described. Children and adolescents with scoliosis more often sought spiritual support as a strategy for coping with a family crisis, while children and adolescents without scoliosis more often used transformation of the situation, passive assessment of the situation, obtaining social support and relying on formal structures. Children and adolescents from both groups used all kinds of strategies, so they have optimal opportunities to cope with a family crisis. Putera et al. assessed the quality of life using the SN-5 scale and strategies of coping with family crisis (F-COPES) among children treated with various therapies due to allergies. They showed that the F-COPES value in the immunotherapy group was 101.56 ± 5.78 (before the test) and reached 105.20 ± 4.31 (after the test; *p* = 0.015), while in the non-immunotherapy group it was 100.36 ± 9.63 (before the test) and 99.96 ± 9.98 (after the test; *p* = 0.224), respectively. The SN-5 value in the immunotherapy group was 30.04 ± 2.78 (before the test) and 11.00 ± 2.33 (after the test; *p* < 0.001), while in the non-immunotherapy group it was 30.04 ± 2.78 (before the test) and 30.04 ± 2.78 (after the test; *p* = 0.767). The researchers did not observe a significant difference in the F-COPES value (*p* = 0.129) [28]. In our own research, after analyzing the results collected using the F-COPES scale of both groups (study and control), it was shown that children and adolescents from the study group more often seek spiritual support (*p* = 0.012), while children and adolescents from the control group more often use such strategies for dealing with a family crisis such as transforming the situation (*p* < 0.001), passive assessment of the situation (*p* < 0.001), gaining social support (*p* < 0.001), and relying on formal structures (*p* = 0.001). Interesting conclusions from research using the F-COPES scale were described by Sari et al. The authors of the publication examined the psychometric properties of the Family Crisis Oriented Personal Assessment Scale (F-COPES) in Turkish society, in a group of carers of people with chronic mental illnesses. Researchers indicated the usefulness of the F-COPES scale in assessing the coping behavior of people taking care of patients with chronic mental illness and noted that it could be used as a measurement tool to assess the correlation with the quality of life [29]. In the research presented in the article, in the group of patients with scoliosis, another significant correlation of F-COPES was noticed in the area of the family’s quality of life. Gaining social support (F-COPES) in the assessment of children and adolescents with SI correlated statistically significantly and negatively only with physical/financial well-being (FQOL).

Children and adolescents with SI, who more often sought social support in solving family problems, assessed the physical and material well-being of the family at a lower level (R = −0.254, *p* = 0.011). Gao et al. showed that in SI patients with a Risser angle of 3–5 and a Cobb angle of 20–40°, Schroth exercises improved HRQOL and stopped the progression of curvature during the conservative treatment period. Both the positioning of the cervical spine, shoulder balance, and HRQOL also improved significantly after using this method by reducing back pain. In the assessment of the researchers and the analysis of the presented results, they proved that SRS-22 improved after treatment (t = 5.578, *p* = 0.013), 4.0 ± 0.3 (t = −3.918, *p* = 0.001), and 3.7± 0.4 (t = −6.468, *p* < 0.001), indicating a positive effect in the treatment process [30]. Our own research showed that the independent predictors of the FQOL level in the area of family interactions in the assessment of children and adolescents with SI included the use of transforming the situation (*p* = 0.019) and relying on formal structures (*p* = 0.004) as a strategy for coping with a family crisis. Statistical analysis also showed that statistically significant (*p* < 0.05) independent predictor of the FQOL level in the area of disability problems was the use of social support. In the group of children without scoliosis, the negative predictor of FQOL was passive assessment of the situation (*p* = 0.008) and obtaining social support (F-COPES). In a cross-sectional study in a group of 104 family caregivers of cancer patients, Mirsoleymani et al. showed, based on the analysis of the results obtained with the use of the F-COPES scale, the importance of their role in better adaptation of patients’ families to a difficult situation [31]. Children and adolescents with SI, who more often sought social support in solving family problems, assessed the physical and material well-being of the family at a lower level (R = −0.254, *p* = 0.011). However, in the assessment of adolescents from the control group, obtaining spiritual support was significantly and positively correlated with emotional well-being (R = 0.207, *p* = 0.039). Ntre et al. studied 143 mothers of children with autism to assess the impact of the presence of a chronic disease in the family on FQOL. Mothers with tertiary education obtained significantly lower total F-COPES scores. The authors showed that passive assessment of the situation was positively correlated with depressive symptoms. Lower results in mobilizing the family to obtain and accept help were associated with more serious disturbances in family life. According to the researchers, mental health professionals should investigate factors that can strengthen strategies for coping with the challenges of having a child with autism [32]. The above-mentioned publications and the presented results indicate the legitimacy of conducting research evaluating the ways and strategies of coping with a family crisis among patients, carers, or families of patients (with chronic diseases) in order to provide them with more individualized and specialized help in the entire process of long-term treatment [33]. Based on the analysis of foreign and domestic literature, it can be concluded that there is a lack of research on the research problem addressed in the article, which is the assessment of F-COPES and its impact on FQOL.

## 5. Conclusions

The results of our study show that factors differentiating the level of FQOL may act as positive or negative predictors in shaping the overall level of FQOL or parts of this variable in the assessment of patients with SI. Adolescents from both compared groups use all possible strategies of coping with crisis situations occurring in the family system. Based on the assessment of children and adolescents with SI, it was shown that the use of the strategy of gaining social support in solving family problems significantly differentiates the level of FQOL. The long process of SI treatment can cause crisis situations (in patients and their families) and affect both the physical and mental health of patients, e.g., by changing the FQOL of their families.

## Figures and Tables

**Table 1:** Characteristics of the study population.

Parameter	Study Group *n* = 100		Control Group *n* = 100		*p*
	Sociodemographic Data				
	*n*	%	*n*	%	
Gender					
Male	17	17%	22	22%	0.475
Female	83	83%	78	78%	
Place of living					
City	74	74%	86	86%	0.052
Village	26	26%	14	14%	
Structure of family					
Two-parent family	100	100%	100	100%	1
Single parent family	0	0%	0	0%	
	M ± SD	Median	M ± SD	Median	0.411
Age of children and adolescents (years)	13.83 ± 1.92	14	14.09 ± 2.16	14	

For quantitative variables, the Mann–Whitney test; for qualitative variables the chi-square test or Fisher’s exact test; *n*- sample size; M—mean value; SD—standard deviation.

**Table 2:** Comparison of the results of the F-COPES scale between children and adolescents from the study and control groups.

Coping Strategies with Family Crisis (F-COPES)	Study Group *n* = 100		Control Group *n* = 100		*p*
	M ± SD	Median	M ± SD	Median	
Transforming the meaning of situation	10.56 ± 2.92	10	12.51 ± 3.43	13	<0.001 *
Passive assessment of the situation	10.78 ± 3.57	11	13.07 ± 3.2	14	<0.001 *
Gaining social support	17.34 ± 5.3	17.5	20.29 ± 3.98	21	<0.001 *
Seeking spiritual support	5.12 ± 2.07	5	4.39 ± 1.72	4	<0.001 *
Relaying on formal structures	4.06 ± 1.59	4	4.77 ± 1.66	4	<0.001 *

Mann–Whitney test; *p*—statistical significance coefficient; M—arithmetic mean; SD—standard deviation; *—*p* < 0.05 indicates a statistically significant relationship.

**Table 3:** Transformation of the meaning of the situation (F-COPES) and passive assessment of the situation versus the level of FQOL in the assessment of children and adolescents with SI.

Level of FQOL	Transformation of the Meaning of the Situation (f-Copes)		Passive Assessment (f-Copes)	
	R	*p*	R	*p*
General FQOL	0.027	0.793	0.027	0.793
Interactions in the family	0.02	0.846	0.02	0.846
Parental functions	−0.085	0.398	−0.085	0.398
Emotional well-being	−0.019	0.854	−0.019	0.854
Physical/financial well-being	−0.035	0.728	−0.035	0.728
Problems with disability	0.167	0.097	0.167	0.097

Spearman’s rank correlation; R—Spearman’s correlation coefficient; *p*—statistical significance coefficient.

**Table 4:** Gaining social support and seeking spiritual support (F-COPES) and the level of FQOL in the assessment of children and adolescents with SI.

Family FQOL	Gaining Social Support (F-COPES)		Seeking Spiritual Support (F-COPES)		Relying on Formal Structures (F-COPES)	
	R	*p*	R	*p*	R	*p*
General FQOL	−0.027	0.789	0.083	0.413	0.061	0.545
Interactions in the family	−0.16	0.113	−0.083	0.413	0.033	0.748
Parental functions	0.074	0.465	−0.015	0.883	0.127	0.209
Emotional well-being	0.099	0.325	−0.002	0.983	−0.03	0.764
Physical/financial well-being	−0.254	0.01 *	−0.021	0.838	0.108	0.285
Problems with disability	−0.175	0.081	0.113	0.264	0.09	0.373

Spearman’s rank correlation; R—Spearman’s correlation coefficient; *p*—statistical significance coefficient; *—value *p* < 0.05 supports the existence of a statistically significant relationship.

**Table 5:** Multivariate analysis of the influence of independent variables on family interactions. Physical and financial well-being (FQOL) assessed by children and adolescents with SI.

Feature	Parameter	95% CI		*p*
**Interactions in the family (FQOL)**				
Transformation of meaning of the situation (F-COPES)	0.429	0.079	0.779	0.019 *
Relying of formal structures (F-COPES)	0.992	0.345	1.639	0.004 *
**Physical/financial well-being (FQOL)**				
Gaining social support (F-COPES)	−0.134	−0.257	−0.011	0.036 *
**Problems with disability**				
Gaining social support (F-COPES)	−0.208	−0.334	−0.083	0.002 *

Multivariate linear regression; *p*—statistical significance coefficient; 95%CI—95% confidence interval; *—*p* < 0.05 indicates a statistically significant relationship.

**Table 6:** Transformation of the meaning of the situation, passive assessment of the situation and gaining social support and the level of FQOL in the assessment of children and adolescents from the control group.

Family FQOL	Transformation of the Meaning of the Situation (F-COPES)		Passive Assessment of the Situation (F-COPES)		Gaining Social Support (F-COPES)	
	R	*p*	R	*p*	R	*p*
General FQOL	−0.001	0.991	−0.04	0.691	−0.013	0.896
Interactions in the family	0.028	0.784	−0.039	0.702	0.039	0.698
Parental functions	−0.026	0.8	0.001	0.991	−0.127	0.21
Emotional well-being	0.001	0.995	−0.157	0.119	−0.103	0.306
Physical/financial well-being	−0.108	0.286	−0.072	0.48	0.014	0.89
Problems with disability	0.194	0.053	0.19	0.058	0.276	0.005 *

Spearman’s rank correlation; R—Spearman’s correlation coefficient; *p*—statistical significance, Coefficient; *—value *p* < 0.05 supports the existence of a statistically significant relationship.

**Table 7:** Seeking spiritual support, relying on formal structures (F-COPES), and the level of FQOL in the assessment of children and adolescents from the control group.

Family FQOL	Seeking Emotional Support (F-COPES)		Relying on Formal Structures (F-COPES)	
	R	*p*	R	*p*
General FQOL	0.188	0.061	0.028	0.779
Interactions in the family	0.094	0.352	0.133	0.187
Parental functions	0.168	0.094	−0.037	0.718
Emotional well-being	0.207	0.039 *	0.128	0.205
Physical/financial well- being	0.045	0.653	0.036	0.719
Problems with disability	0.009	0.932	−0.003	0.979

Spearman’s rank correlation; R—Spearman’s correlation coefficient; *p*—statistical significance. Coefficient; *—value *p* < 0.05 supports the existence of a statistically significant relationship.

**Table 8:** Multivariate analysis of the impact of independent variables on emotional well-being (FQOL) assessed by children and adolescents from the control group.

Feature	Parameter	95% CI		*p*
Passive assessment of the situation (F-COPES)	−0.238	−0.41	−0.066	0.008 *
Gaining social support (F-COPES)	−0.221	−0.393	−0.049	0.014 *
Seeking emotional support (F-COPES)	0.291	0.03	0.552	0.031 *

Multivariate linear regression, *p*—statistical significance coefficient, 95%CI—95% confidence interval, *—*p* < 0.05 indicates a statistically significant relationship.

## Data Availability

Not applicable.
